# Photodynamic Synergistic Effect of Pheophorbide a and Doxorubicin in Combined Treatment against Tumoral Cells

**DOI:** 10.3390/cancers9020018

**Published:** 2017-02-17

**Authors:** Rubén Ruiz-González, Paula Milán, Roger Bresolí-Obach, Juan Carlos Stockert, Angeles Villanueva, Magdalena Cañete, Santi Nonell

**Affiliations:** 1 Institut Químic de Sarrià, Universitat Ramon Llull, Via Augusta 390, 08017 Barcelona, Spain; ruben.ruiz@iqs.url.edu (R.R.-G.); rogerbresolio@iqs.url.edu (R.B.-O.); 2 Departamento de Biología, Facultad de Ciencias, Universidad Autónoma de Madrid, Darwin 2, 28049 Cantoblanco-Madrid, Spain; paula.milan@estudiante.uam.es (P.M.); juancarlos.stockert@gmail.com (J.C.S.); angeles.villanueva@uam.es (A.V.)

**Keywords:** photodynamic therapy, doxorubicin, pheophorbide a, synergic treatment, HeLa cells

## Abstract

A combination of therapies to treat cancer malignancies is at the forefront of research with the aim to reduce drug doses (ultimately side effects) and diminish the possibility of resistance emergence given the multitarget strategy. With this goal in mind, in the present study, we report the combination between the chemotherapeutic drug doxorubicin (DOXO) and the photosensitizing agent pheophorbide a (PhA) to inactivate HeLa cells. Photophysical studies revealed that DOXO can quench the excited states of PhA, detracting from its photosensitizing ability. DOXO can itself photosensitize the production of singlet oxygen; however, this is largely suppressed when bound to DNA. Photodynamic treatments of cells incubated with DOXO and PhA led to different outcomes depending on the concentrations and administration protocols, ranging from antagonistic to synergic for the same concentrations. Taken together, the results indicate that an appropriate combination of DOXO with PhA and red light may produce improved cytotoxicity with a smaller dose of the chemotherapeutic drug, as a result of the different subcellular localization, targets and mode of action of the two agents.

## 1. Introduction

Chemotherapy is the most common strategy applied to treat cancer, with up to 90 types of chemotherapy drugs already approved [1]. It is less invasive than surgery or radiotherapy, so the overall negative impact on the patient is smaller. This type of therapy is very efficient in killing tumor cells and preventing metastasis. Despite these facts, resistance to chemotherapy is the major pitfall that exists in this therapeutic approach. Resistance may be inherent, i.e., from the beginning of treatment, or acquired, appearing after a partial response to the drug. Some of the mechanisms by which resistance to chemotherapy develops include exclusion of the drug from the tumor cells, failure in the activation of the pro-drug to its active form, increased detoxification, altered drug targeting, increased tumor cell repair after injury or escape of the apoptotic response [2,3]. These processes can be caused by the expression of efflux proteins that decrease intracellular levels of the drug (i.e., P-glycoprotein, MRP1), induction of genes encoding anti-apoptotic proteins (i.e., Bcl-2, Bcl-XL), increased DNA repair or the regulation of the expression of biotransformation enzymes [4].

On the other hand, photodynamic therapy (PDT) is a type of non-invasive treatment that involves the use of light-active agents named photosensitizers (PSs), which produce reactive oxygen species (ROS) upon illumination with light of a specific wavelength, causing unspecific cell damage and ultimately killing the cancer cells [5,6]. Among its advantages, PDT enables treating tumors that cannot be removed by surgery, can be applied to vulnerable patients provided its minimal side effects and may help overcome cancer drug resistance [7,8]. Of special interest, and unlike chemotherapeutic drugs, the PS is regenerated in most cases after ROS are produced, which enables it to participate in several therapeutic cycles. The removal of the tumor via PDT depends on the type of PS, its concentration and location, the irradiation wavelength and light dose, the oxygenation levels and the type of ROS generated [6]. From a mechanistic point of view, the action of the initially-formed ROS is localized in the vicinity of the site where the PS has accumulated. Oxidation of biomolecules, such as lipids, amino acids and proteins, can cause irreversible damage to organelles and cell destruction [9]. Cell death can occur via apoptosis, necrosis or autophagy, the final mechanistic outcome being dictated by intracellular PS accumulation [10,11,12]. Most PSs are capable of accumulating in tumor tissue; however, they can also be targeted to the microvessels of the neovasculature that nourishes it (vascular PDT) causing additionally direct and indirect effect to the tumor microenvironment [13,14,15]. Moreover, their photodynamic action also helps to activate the immune system, which can further stimulate tumor suppression (immuno PDT) [16,17,18,19]. Provided that PDT-mediated immune reactions are likely involved in final tumor eradication, combinations of PDT with immunotherapy have been explored, including cytokine therapy, microbial adjuvants or antibodies (photoimmunotherapy (PIT)) [20].

Recent approaches in anticancer therapies include the application of combined chemotherapeutic drugs, delivered together in order to reduce the individual toxicity of each drug (and ultimately side effects) and decrease the likelihood of generating resistance [21,22]. The combination of different anticancer therapies is also being actively explored [23,24] as different cell signaling pathways are simultaneously activated. Hence, tumor cells are destroyed in a more efficient manner by the additive or synergistic effect of both treatments, allowing a reduction in the dose of the most toxic therapeutic agent [23,24].

The combination of a chemotherapeutic drug with a photosensitizing agent is an increasingly growing area of study in vitro, in vivo or even in clinical trials [25]. Several studies have been addressed to evaluate proposed combinations in cell culture models. One of the first ones was conducted by Peterson et al. and evaluated the interaction between doxorubicin and meso-chlorin e_6_ monoethylene diamine (Mce_6_) against human epithelial ovarian carcinoma, showing additive or synergic effects depending on the dose at which each of the therapeutic agents was administered [26]. Datta et al. [27] studied the photodynamic effect of 5-aminolevulinic acid (ALA) in combination with mitomycin C on J82 bladder cell lines, either regular or mitomycin resistant. In this scenario, results on cell viability indicated a higher sensitivity to PDT for the mitomycin-sensitive cell line. Moreover, the combination treatment resulted in an enhanced effect when the chemotherapeutic agent was given first both for parental and mitomycin-sensitive cells [27]. Another study was the application of cisplatin and porfimer sodium (HpD) to L5178Y lymphoma cells where the authors described a marked synergistic effect and increased apoptosis death [28]. In the same line, low doses of cisplatin or gemcitabine in combination with HpD-based or indocyanine-green-based PDT achieved an additive or synergic effect in lung and breast cancer cells, respectively [29,30]. These positive results were complemented by mechanistic evaluation of the treatment responses. Combining sodium talaporfin with oxaliplatin, cis-diaminodichloroplatinum or gemcitabine to inactivate NOZ gallbladder carcinoma cells, there appears in all cases a synergistic result in the combined treatments with respect to the inactivation produced by each of the drugs separately [31]. These treatments not only significantly increase cell inactivation, but also reduce the concentrations of PS and, more importantly, the antineoplastic agent as described in HpD and cisplatin treatments in mouse cancer cells [32]. The use of ALA in combination treatments with doxorubicin or vincristine in murine leukemic cells does not always produce an additive effect, but avoids the multiresistance of cells to treatments [33]. However, in some combinations of both therapies, there may be produced not a synergic, but antagonistic effect, as described for erlotinib or cetuximab when applied in combination with PDT using meso-tetraphenylporphine [34]. With the aim to achieve better selectivity, approaches combining chemotherapy with PIT have also been conducted. Combination effects have been assessed in human ovarian cancer cell lines for the addition of chemotherapeutic agents (cisplatin or 2,5-bis(5-hydroxymethyl-2-thienyl)furan (SOS thiophene) and chlorin PSs linked to antibody fragments against different carcinoma antigens with promising results [35,36]. In the same line, Rizvi et al. achieved the reduction of chemotherapeutic cycles when including EGFR-targeted Mce_6_ PIT for an ovarian carcinoma murine model [37].

**Figure 1:**
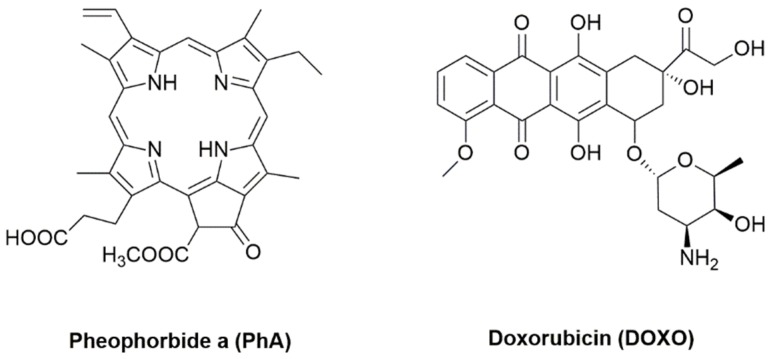
Structures of the active compounds studied in this work.

In the present study, we have analyzed the effect on the cell survival of a photosensitizing compound (pheophorbide a (PhA)) in combination with the traditional antineoplastic agent doxorubicin (DOXO) (Figure 1). PhA is a photosensitive chlorophyll metabolite with immunostimulation activity [38], which at adequate concentrations causes the apoptosis of tumor cells [39]. Despite its negligible toxicity to healthy cells in the dark, exposure to light elicits apoptosis, cell cycle arrest at sub-G1, abolition of antiapoptotic protein Bcl-2, cytochrome-C release to the cytosol and activation of procaspases 3 and 9 [40]. PhA has been shown to induce apoptosis in Jurkat leukemia cells [41], human hepatocellular carcinoma [42] or uterine carcinosarcomas [43] in photodynamic treatments. On the chemotherapeutic side, DOXO is a nonselective anthracycline antibiotic class I whose mechanism of action is the inhibition of enzymes responsible for DNA replication. This drug intercalates in the DNA double helix and interferes with topoisomerases I and II, thereby preventing the binding of the DNA strands and leading to cell death [44]. DOXO is often used in the treatment of solid tumors; however, it has significant side effects, such as cardiomyopathies [45].

## 2. Results

A potential problem arising from the combination of a PS and a chemotherapeutic drug is the interaction between the two, which could result in the quenching of the excited states of the PS, thereby detracting from its photodynamic activity. This was assessed by a series of photophysical experiments described below.

### 2.1. Photophysical Studies

#### 2.1.1. Absorption and Fluorescence

**Figure 2:**
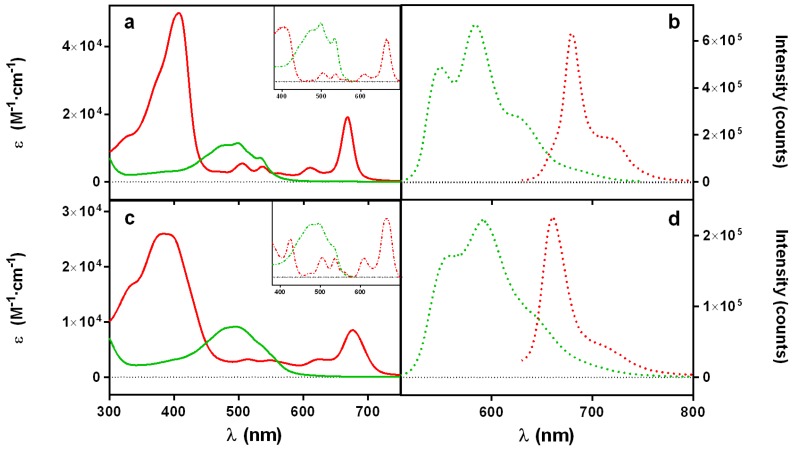
Absorption (**a**,**c**) and fluorescence (**b**,**d**) spectra of DOXO (green) and PhA (red) in EtOH (**a**,**b**) and water (**c**,**d**). DOXO was excited at 455 nm and PhA at 610 nm. Insets: (**a**,**c**) excitation spectra of the fluorescence at 705 nm for PhA and 610 nm for DOXO.

As a first approach, we measured the absorption and fluorescence spectra of solutions of DOXO and PhA alone in two different solvents, namely ethanol (EtOH) and water, taken as simple models of the cell membrane and the cytoplasm, respectively (Figure 2). In EtOH, both the absorption and fluorescence spectra show narrow structured bands, and the fluorescence excitation spectra match the corresponding absorption spectra (Figure 2a,b). These observations indicate that both compounds exist as monomers. In contrast, the absorption spectra in water (Figure 2c) show broader and non-structured bands, red-shifted in the case of DOXO and blue-shifted for PhA (Table 1). Notwithstanding, the fluorescence emission (Figure 2d) and excitation (Figure 2c, inset) spectra are very similar to those recorded in EtOH, although much weaker in intensity, particularly for PhA. These findings indicate that both compounds exist as a mixture of fluorescent monomers and essentially non-fluorescent aggregates in aqueous solutions (J-type in the case of DOXO and H-type PhA).

**Figure 3:**
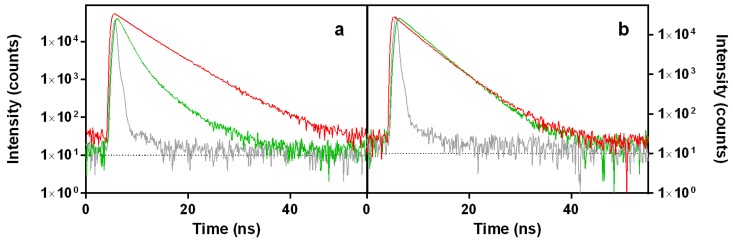
Time-resolved fluorescence (TRF) decays for DOXO (green) and PhA (red) in EtOH (**a**) and D_2_O (**b**). Grey traces represent the instrument response function. DOXO was excited at 504 nm and PhA at 654 nm. Observation wavelengths were 660 nm and 710 nm, respectively.

Figure 3 shows the fluorescence decays for DOXO (green traces) and PhA (red traces). In EtOH, DOXO decays with a biexponential function with time constants of 1.4 ns (93% weight) and 4.3 ns (7% weight). In D_2_O, however, only one lifetime (4.0 ns) was needed to adequately fit the decay. On the other hand, PhA shows a monoexponential decay in EtOH with the lifetime of 5.9 ns, whereas the weak emission in D_2_O showed two components with lifetimes of 4.8 ns (61% weight) and 2.6 ns (31% weight).

**Figure 4:**
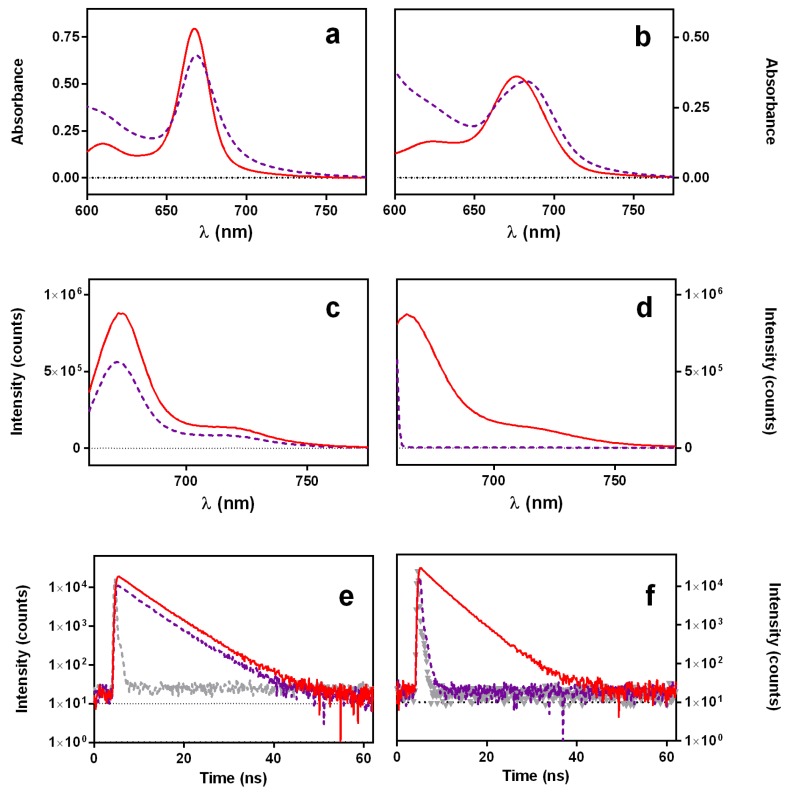
Absorption spectra (**a**,**b**), fluorescence spectra (**c**,**d**) and fluorescence decay (**e**,**f**) of PhA 25 μM alone (red solid lines) and in the presence of 1 mM DOXO (dashed violet lines) in EtOH (left panels) and H_2_O (right panels) solutions. For TRF measurements, λ_exc_ = 654 nm and λ_obs_ = 750 nm. Grey dashed traces correspond to the instrument response function.

In separate experiments, we recorded the absorption and fluorescence spectra and the fluorescence decay of aqueous and ethanolic solutions of PhA in the presence of 1 mM DOXO (Figure 4). In both solvents, the absorption bands of PhA broadened and decreased their amplitude (Figure 4a,b). On the other hand, the shape of the fluorescence spectra and the decay lifetime were unaffected by the addition of DOXO, but the fluorescence intensity decreased by approximately 40% in EtOH and almost completely in water (Figure 4c–f). These observations indicate that the PhA and DOXO form a ground-state complex that causes static quenching of the PhA singlet excited state.

#### 2.1.2. Singlet Oxygen Photosensitization by Doxorubicin

**Figure 5:**
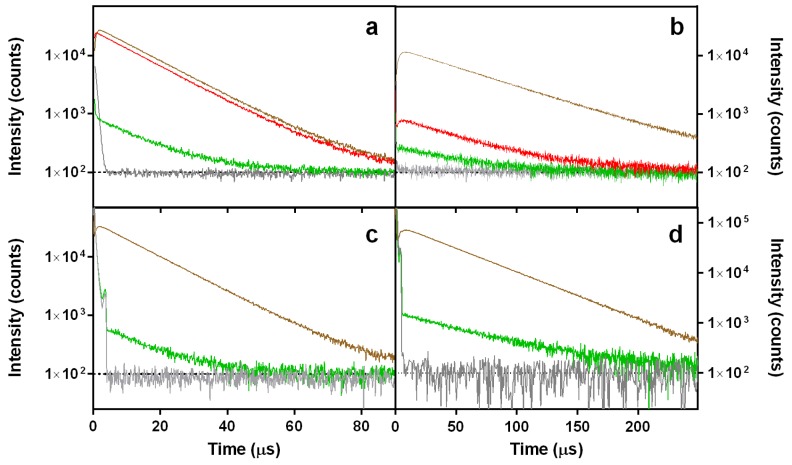
Singlet oxygen phosphorescence kinetics at 1275 nm in EtOH (**a**,**c**) and D_2_O (**b**,**d**) solutions of DOXO (green), PhA (red) and reference photosensitizers (PSs) (brown). The signals completely disappeared in the presence of 25 mM NaN_3_, a typical ^1^O_2_ quencher (grey). Rose Bengal (RB) in EtOH and flavin mononucleotide (FMN) in D_2_O were used as references. The excitation wavelength was 532 nm for (**a**,**b**) and 473 nm for (**c**,**d**).

The strong fluorescence of DOXO suggests that it might generate ROS upon exposure to light. Its capacity to photosensitize ^1^O_2_ was assessed in D_2_O and EtOH by time-resolved detection of the near-infrared ^1^O_2_ phosphorescence at 1275 nm. The luminescence observed is assigned to ^1^O_2_ because the transient is completely quenched in presence of sodium azide (NaN_3_, a well-known ^1^O_2_ quencher; 25 mM) [46]. As shown in Figure 5, DOXO does indeed generate ^1^O_2_ with quantum yield Φ_Δ_ = 0.03 in EtOH and Φ_∆_ = 0.01 in D_2_O, irrespective of the excitation wavelength. The decrease of Φ_Δ_ in D_2_O is consistent with the occurrence of aggregation in this solvent.

#### 2.1.3. Interaction between Doxorubicin and DNA

**Figure 6:**
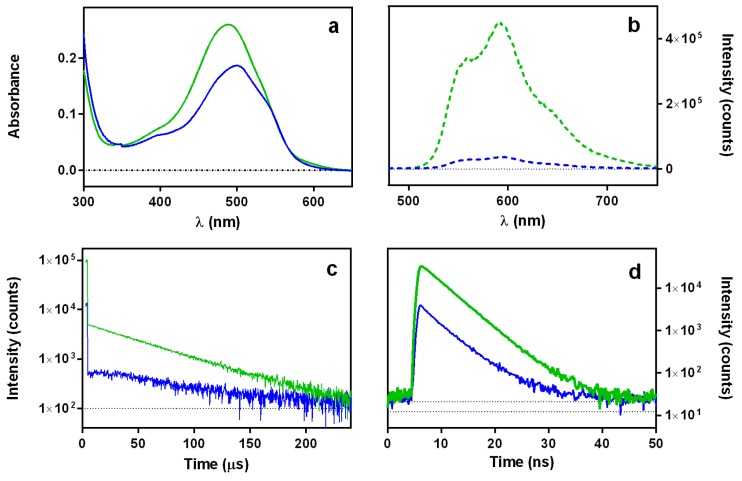
Spectroscopic and photophysical properties of DOXO in D_2_O solutions in the absence (green traces) and presence (blue traces) of 60 μg/mL DNA: absorption spectra (**a**); fluorescence emission spectra (**b**); singlet oxygen phosphorescence kinetics at 1275 nm upon excitation at 473 nm (**c**); and fluorescence kinetics upon excitation at 504 nm and observation at 660 nm (**d**).

DOXO is well known to localize in the nucleus where it binds to DNA. Spectral and photophysical consequences of DNA binding are shown in Figure 6: (i) the absorption spectrum appears more structured (Figure 6a); (ii) the fluorescence is strongly quenched (Figure 6b); (iii) the amount of ^1^O_2_ produced is strongly reduced (Figure 6c); and (iv) the triplet lifetime is extended, as demonstrated by the slower rise of the ^1^O_2_ phosphorescence signal. On the other hand, the fluorescence lifetime does not change (Figure 6d). These observations are consistent with strong static quenching of the DOXO excited states and shielding from oxygen upon binding to DNA.

### 2.2. Subcellular Localization Studies

**Figure 7:**
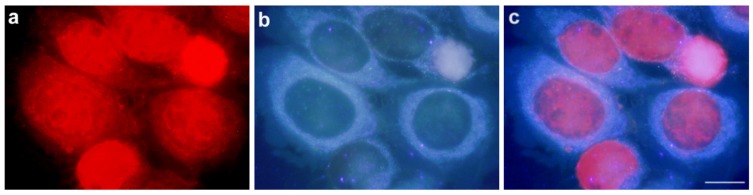
HeLa cells treated with 0.2 μM DOXO for 24 h and observed under fluorescence microscope. (**a**) Cells observed under green excitation; (**b**) the same field observed under UV excitation; (**c**) overlapping images from (**a**) and (**b**). Scale bar: 10 μM.

Figure 7 shows fluorescence microscopy images of HeLa cells incubated with 0.2 μM DOXO and observed immediately after 24 h of incubation. In (a), fluorescence arising from DOXO is imaged upon green light excitation. In (b), cell’s autofluorescence spread over the cytoplasm upon UV-light irradiation is observed for the same set of cells. Finally, (c) is an overlap of previous panels, which unambiguously allows localizing DOXO in the cell nucleus.

**Figure 8:**
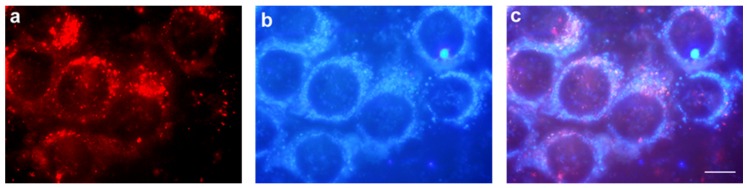
HeLa cells treated with 2 μM PhA for 4 h observed in fluorescence microscopy. (**a**) Cells observed under green light excitation; (**b**) the same field observed under UV light; (**c**) overlapping images from (**a**) and (**b**). Scale bar: 10 μM.

Fluorescence images of HeLa cells incubated for 4 h with 2 μM PhA are depicted in Figure 8. In (a), mitochondria autofluorescence is observed upon UV excitation. (b) shows the PhA fluorescence distributed in granules of variable size through the cytoplasm. The superposition of the two images (c) rules out mitochondrial localization in HeLa cells.

**Figure 9:**
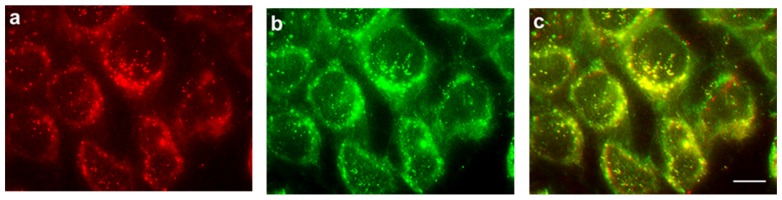
HeLa cells treated with 2 μM PhA and 50 nM LysoTracker Green for 4 h, observed by fluorescence microscopy. (**a**) PhA fluorescence observed under green light excitation; (**b**) LysoTracker Green fluorescence under blue light excitation; (**c**) merged images from (**a**) and (**b**). Scale bar: 10 μM.

In additional experiments, the commercial fluorescent probe LysoTracker was co-incubated with PhA in HeLa cells under the same experimental conditions. Figure 9 shows the fluorescence of PhA (a), the fluorescence of the lysosomal marker (b) and the superposition of the two images (c). There is an almost perfect overlap between the PhA fluorescence and that of the lysosomal marker.

### 2.3. Cell Viability Studies

In order to reveal the effects of combined photodynamic and chemotherapeutic treatments, cell viability studies by 3-(4,5-dimethylthiazol-2-yl)-2,5-diphenyl-2H tetrazolium bromide (MTT) were carried out with the individual components aiming at identifying the conditions leading to 50% cell death for each component.

#### 2.3.1. Treatments with Doxorubicin

**Figure 10:**
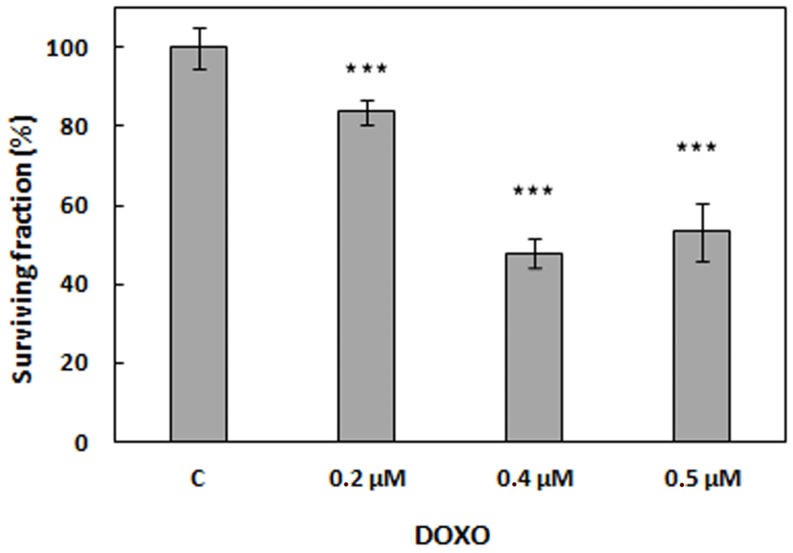
Viability of HeLa cells treated with DOXO for 24 h at different concentrations and non-treated control cells (c). *** *p* < 0.001. The standard deviation (SD) is the average of at least three experiments.

HeLa cells were incubated with different DOXO concentrations, and cell viability was assessed 24 h after treatment. As shown in Figure 10, concentrations of 0.4 μM and 0.5 μM produced a cell inactivation of approximately 50%. Thus, the lowest concentration (0.4 μM) was chosen for combination therapies.

Given the ability of DOXO to produce ^1^O_2_ upon exposure to light, we assessed whether it could be used as a dual agent, namely cytotoxic and photocytotoxic. Thus, in separate experiments, cells were pre-incubated with 0.2 μM and 0.4 μM DOXO and irradiated with green light. Cell viability of irradiated samples was identical to that shown in Figure 10.

#### 2.3.2. Treatments with Pheophorbide a

**Figure 11:**
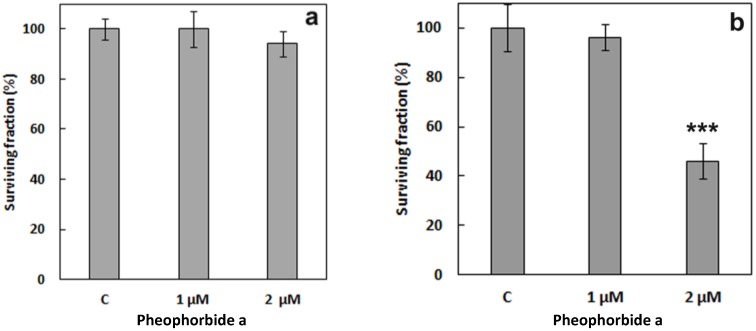
Viability of HeLa cells treated with PhA at different concentrations and non-treated control cells (c) for 4 h in the dark (**a**) and after irradiation (**b**). *** *p* < 0.001. SD is the average of at least three experiments.

Figure 11 shows viability results on HeLa cells incubated with 1 μM and 2 μM PhA for 4 h. In the absence of light (Figure 11a), the surviving fraction of treated cells was similar to that of control cells. On the contrary, after 4 h of incubation with 2 μM PhA and 15 min of red-light irradiation (6.4 J/cm^2^), the fraction of surviving cells had dropped to approximately 50% (Figure 11b). Irradiation of control cells in the absence of PhA did not produce any measurable cytotoxicity. Hence, the above conditions were chosen for the combination experiments.

#### 2.3.3. Combined Treatment of Doxorubicin and Pheophorbide a

For the combination assays, the concentrations of DOXO and PhA that individually caused 50% cell death each were chosen (2 μM PhA and 0.4 μM DOXO). The timing of the chemo- and photo-therapeutic events is expected to play a major role in the outcome of the combined treatments. In order to identify the best synchronization conditions, the two compounds were administered according to three different protocols: In the first protocol (termed PhA-DOXO), cells were incubated with PhA for 4 h, washed to remove any unbound PhA, irradiated for 15 min and then further incubated with DOXO for a period of 24 h. In the second protocol (DOXO-PhA), cells were first treated with DOXO for 24 h, washed, incubated with PhA for 4 h, washed again and finally irradiated for 15 min. In the third protocol (DOXO + PhA), cells were incubated with DOXO for 20 h, washed, co-incubated with PhA and DOXO for 4 h, washed again and finally irradiated for 15 min. At the end of the three protocols, cells have been incubated with DOXO for 24 h and with PhA for 4 h, and the same light fluence has been delivered.

**Figure 12:**
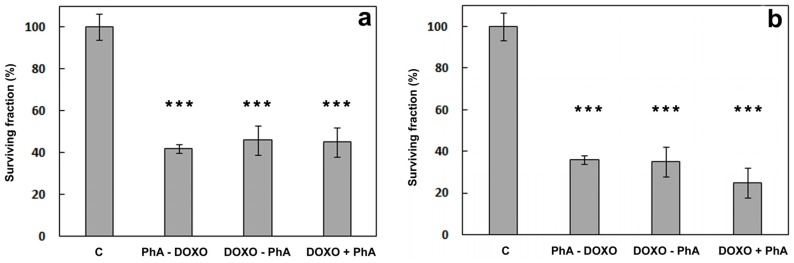
Viability of HeLa cells treated with DOXO 0.4 μM and PhA 2 μM according to three different protocols. Samples were either kept in the dark (**a**) or irradiated for 15 min after PhA incubation (**b**). c: non-treated control cells. PhA-DOXO: cells incubated with PhA for 4 h, washed and incubated with DOXO for 24 h. DOXO-PhA: cells incubated with DOXO for 24 h, washed and incubated with PhA for 4 h. DOXO + PhA: cells incubated with DOXO for 20 h, washed and co-incubated with PhA and DOXO for 4 h. *** *p* < 0.001. SD is the average of at least three separate experiments.

Figure 12a shows the viability of the cells after delivery of the two compounds according to three different protocols in the absence of light. The combination of DOXO with PhA does not increase their intrinsic dark toxicity (cf. Figure 10 and Figure 11a). Cell viability data after irradiation are shown in Figure 12b. While one could have expected a cell survival no higher than 25% (50% DOXO × 50% PhA and light, if additive), this outcome was only observed with the third protocol (DOXO + PhA).

**Figure 13:**
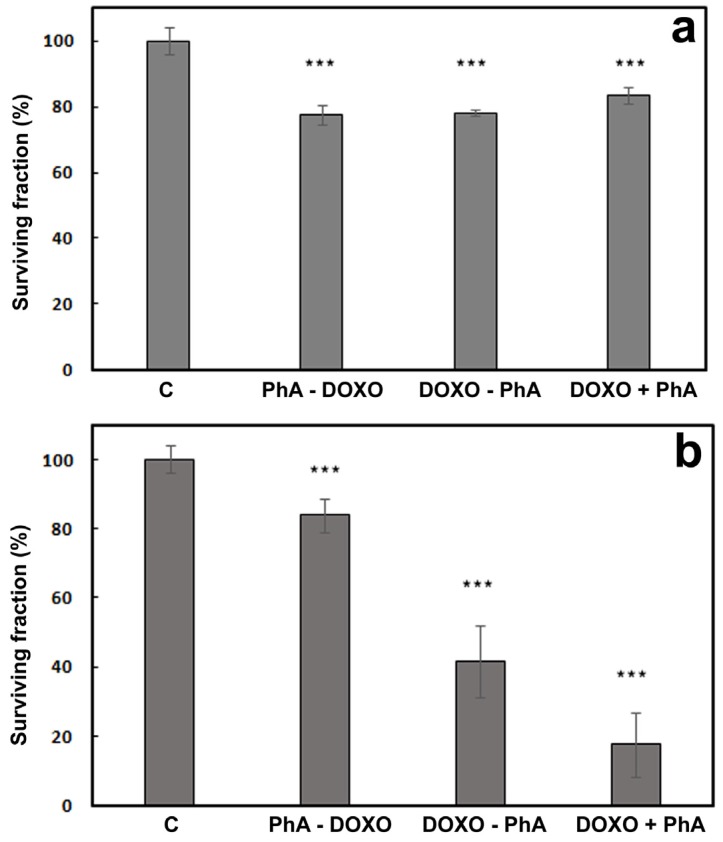
Viability of HeLa cells treated with DOXO 0.2 μM and PhA 2 μM according to three different protocols. Samples were either kept in the dark (**a**) or irradiated for 15 min after PhA incubation (**b**). c: non-treated control cells. PhA-DOXO: cells incubated with PhA for 4 h, washed and incubated with DOXO for 24 h. DOXO-PhA: cells incubated with DOXO for 24 h, washed and incubated with PhA for 4 h. DOXO + PhA: cells incubated with DOXO for 20 h, washed and coincubated with PhA and DOXO for 4 h. *** *p* < 0.001. SD is the average of at least three separate experiments.

The results above indicate that the combination of a PhA photodynamic treatment with DOXO chemotherapy is sub-additive for the PhA-DOXO and DOXO-PhA protocols. In order to better assess the role of the photodynamic component in the combination treatments, the concentration of DOXO was lowered down to 0.2 μM, maintaining invariable all other experimental conditions. At this DOXO concentration, cell viability was 84% (Figure 10). The results of this new set of experiments are shown in Figure 13.

In this case, there was a remarkable difference in outcome between the three protocols. For PhA-DOXO, there was no appreciable difference in cell viability between the dark and light experiments. For DOXO-PhA, the expected additive effect was observed (0.84 × 0.46 = 0.39, which compares well with the observed 42%). Remarkably, the DOXO + PhA treatment resulted in a survival fraction of 18%, which indicates that a synergistic effect can indeed be achieved under the proper conditions.

## 3. Discussion

Chemotherapy is the most common treatment used for fighting cancer malignancies despite its major problems, such as high toxicity and the onset of resistance [2]. Therapeutic alternatives are being highly explored, such as combined treatments using drugs of various kinds aiming at reducing the dose of the toxic chemotherapeutic agent; therefore, the side effects and the acquisition of resistance [28]. PDT is a local treatment whose mode of action does not usually cause the emergence of resistance or the inhibition of the immune system. Rather, it even activates the immune system [47], which makes PDT a potential partner for combined treatments [16]. In this paper, we set out to study combinations of PhA, a representative PS, with DOXO, a well-established chemotherapeutic drug [48], to assess whether such combinations would allow reducing the concentration of the toxic DOXO.

DOXO’s properties and mode of action have been extensively characterized. It accumulates in the nucleus (Figure 7), where it exerts its damage as a topoisomerase inhibitor [44], and its 1,4-dihydroxyanthraquinone chromophore is fluorescent under green light irradiation [49,50]. On the other hand, anthraquinones are known to produce ROS through electron transfer processes with biological molecules [51,52,53,54] and can also generate ^1^O_2_ under irradiation [55,56]. Indeed DOXO generates ^1^O_2_ with quantum yields Φ_∆_ = 0.03 in EtOH and Φ_∆_ = 0.01 in D_2_O as a consequence of its aggregation. However, HeLa cells incubated with 0.2 μM and 0.4 μM DOXO and irradiated with green light did not show any increased toxicity compared to the non-irradiated samples. 1,4-dihydroxyanthraquinone derivatives efficiently bind to DNA [57,58,59], which causes a marked fluorescence loss [60,61]. We have confirmed that this is the case also for DOXO (Figure 6) and have assessed that it leads to a 10-fold reduction in its capacity to produce ^1^O_2_ by photosensitization.

PhA is a chlorophyll-based metabolite endowed with appealing photophysical properties, such as strong absorption in the red part of the spectrum, red fluorescence and a high capacity to generate ^1^O_2_ when it is in monomeric form (Table 1). Our values are in excellent agreement with previous literature reports (Φ_∆_ = 0.59 in EtOH; Φ_∆_ < 0.02 in water) [62,63]. PhA has been evaluated as a PDT agent against different cancer malignancies [40,64]. In our study, PhA localizes in lysosomes (Figure 8). This is in line with the reports that PhA methyl ester localizes in endoplasmatic reticulum/Golgi and lysosomes [65]. Lysosomes have been previously shown to be a good target for triggering apoptosis in PDT treatments [66,67].

It was hypothesized that the combined use of drugs with different cellular localizations could increase their cytotoxic capacity and could hence be a valuable strategy to reduce their concentrations and thus their side effects [68]. The combination of PhA with DOXO seemed suitable, as damage would occur in the lysosomal compartment and the nucleus, respectively. In this sense, in vitro combinations of DOXO with different PSs and cell lines have already been explored aiming at enhancing the final cell-killing effect [26,69,70,71,72]. We determined the IC_50_ of DOXO in HeLa cells (0.4 μM) after an incubation time of 24 h. Likewise, we found that 15 min red-light irradiation of HeLa cells incubated for 4 h with PhA 2 μM induced also 50% cell death with a lack of toxicity in the absence of light. Combination implies sequencing of the active agents, and thus, the order is a significant factor. Indeed, it has been reported that synergic interaction between PDT and chemotherapy is dependent not only on the nature of the PS and the chemotherapeutical agent, as well as on the irradiance dose, but also on the treatment sequence [27,69,71,73]. On the one hand, we have previously mentioned that Datta et al. found that combined treatment of ALA-PDT and mitomycin C resulted in an enhanced effect when the chemotherapeutic agent was given first both for parental and mitomycin-sensitive cells [27]. On the other hand, Cowled et al. showed that, when DOXO was administered with HpD and at the time of irradiation, the photodynamic effect was potentiated, whereas DOXO administration was less effective after PDT, both in vivo [69]. In this line, Kirveliene et al. showed that combined treatment of DOXO and temoporfin was more effective if DOXO was applied right after light exposure in murine hepatoma in vitro and in vivo [71]. As a final example, Rizvi et al. observed synergism in benzoporphyrin-based treatment followed by low-dose carboplatin against ovarian cancer cells grown in a three-dimensional model, which was not achieved with the reverse treatment order [73]. Hence, the combined effect of DOXO and PhA + light on HeLa cell viability was explored for 0.4 μM DOXO and 2 μM PhA using three different delivery protocols.

The outcome of the combined treatments was evaluated according to the method described by Valeriote and Lin [74], which compares the efficacy of the individual drugs (ε_A_, ε_B_) with that of its combination (ε_A+B_). The treatment is synergic if ε_A+B_ < (ε_A_ × ε_B_)/100, additive, if ε_A+B_ = (ε_A_ × ε_B_)/100, sub-additive if (ε_A_ × ε_B_)/100 < ε_A+B_ < ε_A_, provided ε_A_ < ε_B_, interference, if ε_A_ < ε_A+B_ < ε_B_, when ε_A_ < ε_B,_ and antagonistic, if ε_B_ < ε_A+B_, when ε_A_ < ε_B_.

Out of the three experimental conditions tested, only DOXO + PhA combined photo-treatment showed better efficacy (25% cell survival) than the individual treatments DOXO 0.4 μM (48%) or PhA + light (46%). This treatment is therefore additive, unlike other combined photodynamic and chemotherapeutic treatments reported in the literature [25,31]. The other two treatments are sub-additive, perhaps due to the quenching of PhA excited states by DOXO. Previous works have observed that DOXO can prevent drug accumulation in combination treatments [71,75]; however, this is not the case provided that the overall result was the same either with DOXO delivered before (DOXO-PhA) or after PhA (PhA-DOXO). We hypothesize that, in addition, the toxicity of 0.4 μM DOXO was so large that it masked the effects of the photodynamic component elicited by PhA. Indeed, using a lower concentration of DOXO (0.2 μM), we found that PhA-DOXO was now borderline between interference and antagonistic; DOXO-PhA was additive; and PhA + DOXO was synergic.

In agreement with previous studies, it can be concluded that not only the relative concentration, but also the order in which the chemotherapeutic and the photodynamic treatments are delivered play key roles in the outcome of DOXO and PhA + light combined treatments in HeLa cells.

## 4. Materials and Methods

### 4.1. Chemicals

Phosphate buffer saline (PBS), doxorubicin (DOXO), deuterium oxide (D_2_O), DNA sodium salt from calf thymus, sodium azide (NaN_3_), 3-(4,5-dimethylthiazol-2-yl)-2,5-diphenyl-2H tetrazolium bromide (MTT) and the photosensitizing agents pheophorbide a (PhA) and Rose Bengal (RB) were purchased from Sigma-Aldrich, Chemical Co. (St. Louis, MO, USA). LysoTracker Green DND-26 was purchased from Thermo Fisher (Waltham, MA, USA). Flavin mononucleotide (FMN) was from Chemodex Ltd. (St. Gallen, Switzerland).

### 4.2. General Spectroscopic Measurements

All spectroscopic measurements were carried out in 1-cm quartz cuvettes (Hellma, Germany) at room temperature. Absorption spectra were recorded on a Cary 6000i spectrophotometer (Varian, Palo Alto, CA, USA). Fluorescence emission spectra were recorded using a Spex Fluoromax-4 spectrofluorometer (Horiba Jobin-Ybon, Edison, NJ, USA). Fluorescence decays were recorded with a time-correlated single photon counting system (Fluotime 200, PicoQuant GmbH, Berlin, Germany) equipped with a red sensitive photomultiplier. Excitation was achieved by either a 504-nm picosecond LED or a 654-nm laser working at a 10-MHz repetition rate. The counting frequency was kept always below 1%. Fluorescence decays were analyzed using the PicoQuant FluoFit v4.6.5 data analysis software. For ^1^O_2_ phosphorescence measurements, an AO-Z-473 solid state AOM Q-switched laser (Changchun New Industries Optoelectronics Technology Co., Changchun, China) working at a 4-kHz repetition rate (<1.5 mW average power) was used for excitation at 473 nm, and a diode-pumped Nd:YAG laser (FTSS355-Q, Crystal Laser, Berlin, Germany) working at a 1-kHz repetition rate (1.2 μJ per pulse, 1-ns pulse-width) was used for excitation at 532 nm. A 1064-nm rugate notch filter (Edmund Optics Ltd., York, UK) was placed in the laser path to remove any NIR emission. The luminescence emitted from the sample was collected at 80 degrees, filtered by a long-pass filter with the cut-off at 1100 nm in order to remove any scattered laser radiation and by a narrow bandpass filter at 1270 nm to isolate the NIR emission. A TE-cooled near-IR sensitive photomultiplier tube assembly (H9170−45, Hamamatsu Photonics Hamamatsu City, Shizuoka Prefacture, Japan) in combination with a multichannel scaler (NanoHarp 250, PicoQuant Gmbh, Berlin, Germany) was used as the photon-counting detector [76]. ^1^O_2_ decays were analyzed using the GraphPad Prism 5.0 software to fit the data to Equation (1).

(1) $$S\left(t\right)=S_{0}\;\times \;\frac{\tau_{∆}}{\tau_{∆}-\tau_{T}}\;\times \;\left(exp\left(-t/\tau_{∆}\right)-exp\left(-t/\tau_{T}\right)\right)$$

where *S*_0_ is a quantity proportional to the singlet oxygen quantum yield, Φ_∆_. For the determination of Φ_∆_ values, optically-matched solutions of DOXO, PhA and suitable reference PSs (Flavin mononucleotide (FMN), Φ_∆_ = 0.56 for D_2_O and Rose Bengal (RB), Φ_∆_ = 0.75 for EtOH [55,56]) were excited and their *S*_0_ values scaled using Equation (2).

(2) $$\phi_{∆,Sample}\;=\;\phi_{∆,\;Ref}\;\times \;\frac{S_{0,Sample}}{S_{0,Ref}}$$

### 4.3. Cell Cultures

Cell cultures from cervical adenocarcinoma human uterus (ATCC^®^ CCL2™, LGC Standards S.L.U., Manassas, VA, USA) HeLa cells were used [77]. The cells were grown with minimum essential medium modified by Dulbecco (DMEM) to which 10% (*v*/*v*) fetal bovine serum, 50 units/mL penicillin, 50 μg/mL streptomycin and 1% (*v*/*v*) 0.2 M *L*-glutamine were added. All of these products were supplied by Invitrogen. Cell were cultured in a 200 SteriCult (Hucoa-Erlöss, Thermo Fisher) incubator with humid atmosphere at 37 °C and 5% CO_2_. Cells were seeded in 25-cm^2^ flasks (F 25) or in multi-well plates (6 or 24 wells rack) from Corning Inc. (New York, NY, USA). Cell manipulation was carried out in a vertical laminar flow hood SUPACRIS 12 (Labconco, Kansas City, MO, USA).

### 4.4. Doxorubicin and Pheophorbide a Subcellular Localization

To characterize the subcellular localization of drugs used in this study, observations on live cells in culture were made in fluorescence microscopy. Immediately after incubation with DOXO or PhA, cells on coverslips were washed 3 times with PBS and mounted on a slide, removing excess liquid. In the case of treatment with PhA plus co-localization, experiments using LysoTracker Green were performed. The probe was prepared at a concentration of 50 nM in complete medium without serum. Cells previously treated with PhA for 4 h were incubated with LysoTracker Green for 5 min, washed with PBS (×3) and observed in fluorescence microscopy.

Microscopic observations and photographs were performed with an Olympus photomicroscope IMT-2, equipped with a mercury lamp HBO 100 W and the corresponding filter sets for fluorescence microscopy: UV (365–390 nm), blue (450–490 nm) and green (510–550 nm). The images were processed using Adobe Photoshop 5.0 software (Adobe Systems, Inc., San José, CA, USA). The routine observation of cells was performed on an inverted Olympus microscope X31 CK (Barcelona, Spain).

### 4.5. Chemotherapeutic, Photodynamic and Combined Treatments

For photodynamic treatments, a stock solution of PhA was prepared at a concentration of 10 mM in DMSO and stored at −20 °C. The PS was diluted in culture medium to the final desired concentration, added to the cells and incubated for 4 h. The wells were washed 3 times with PBS, and fresh culture medium was added prior to irradiation. Then, they were irradiated for 15 min with red light from an LED Par 64 Short V2 lamp (Showtec, Kerkrade, The Netherlands) at 632 nm and 7.1 mW/cm^2^. After photodynamic treatments, the plates were kept in the incubator until further processing.

For chemotherapeutic treatments, a 100 mM stock solution of DOXO in water was prepared and stored at −20 °C. Cells were incubated for 24 h with DOXO previously diluted in culture medium, washed three times with PBS and left with culture medium in the incubator until further processing.

For combined treatments, DOXO and PhA were delivered according to three different protocols as described in Section 2.3.3.

### 4.6. Cell Viability

Evaluation of cell survival was performed 24 h after each treatment, using the MTT assay. A 1-mg/mL stock solution of MTT in water was prepared and filtered. From this initial solution, a 0.05-mg/mL dilution was prepared in culture medium, and a 0.5-mL aliquot of this dilution was added to each well of a 24-well rack. After 3 h, DMSO was added to dissolve the formazan precipitate. The absorbance of formazan was then measured in a plate reader (Tecan Spectra Fluor, MTX Lab Systems, Bradenton, FL, USA) at 540 nm. The survival of the cells was represented as the percentage absorbance of the treated cells with respect to the absorbance of control cells.

### 4.7. Statistical Analysis

Statistical analyses were performed using the R 3.2.5 program. By ANOVA, test the mean factor experiments (each condition vs. control) were compared. It was considered that there were significant differences between means when: * *p* < 0.05; ** *p* < 0.01 and *** *p* < 0.001.

## 5. Conclusions

Our study supports the trend towards the development of combination therapies by evaluating the combined use of the photosensitizing agent PhA and the chemotherapeutic drug DOXO to kill HeLa cells. Both compounds show different subcellular accumulation in this cell line and exert damage by different modes of action.

Photophysical studies of the compounds alone and in combination have revealed that DOXO quenches the excited states of PhA, detracting from its photosensitizing ability. On the other hand, while DOXO can itself photosensitize the production of ^1^O_2_, this is largely suppressed by aggregation and by binding to DNA.

Three different combined treatment protocols have been assayed. The order of drug and light delivery plays a crucial role in the final outcome of the treatments, ranging from antagonistic to synergic for the same concentrations, which is supported by several previous reports. Thus, while combined photodynamic and chemotherapeutic treatments show strong potential to decrease the dose of the toxic chemotherapeutic agent and therefore its side effects, the delivery protocols must be very finely tuned to achieve this desired outcome.

## Figures and Tables

**Table 1:** Optical and photochemical properties of the compounds studied in this work.

Compound	Solvent	λ_Abs_/nm ^a^	λ_Fluo_/nm ^b^	τ_F1_/ns ^c^	τ_F2_/ns	Φ_Δ_
**DOXO**	EtOH	497	582	1.4 (0.93)	4.3 (0.07)	0.03 ^d,e^
	D_2_O	489	590	4.0	-	0.01 ^d,e^
**PhA**	EtOH	668	674	5.9	-	0.61 ^d^
	D_2_O	682	666	4.8 (0.61)	2.6 (0.39)	0.04 ^d^

^a^ Maximum of intensity for DOXO and maximum of the lowest energy absorption band for PhA; ^b^ maxima of the most intense fluorescence band; ^c^ values in brackets are the fractional amplitudes; ^d^ λ_exc_ = 532 nm; ^e^ λ_exc_ = 473 nm.
